# The Influence of Naringin or Hesperidin Dietary Supplementation on Broiler Meat Quality and Oxidative Stability

**DOI:** 10.1371/journal.pone.0141652

**Published:** 2015-10-28

**Authors:** Michael Goliomytis, Nikos Kartsonas, Maria A. Charismiadou, George K. Symeon, Panagiotis E. Simitzis, Stelios G. Deligeorgis

**Affiliations:** Department of Animal Science and Aquaculture, Agricultural University of Athens, Athens, Greece; Seoul National University, REPUBLIC OF KOREA

## Abstract

An experiment was conducted to examine the effects of supplementing broiler feed with hesperidin or naringin, on growth performance, carcass characteristics, breast meat quality and the oxidative stability of breast and thigh meat. Two hundred and forty 1-day-old Ross 308 broiler chickens were randomly assigned to 6 groups. One of the groups served as a control (C) and was given commercial basal diets, whereas the other five groups were given the same diets further supplemented with naringin at 0.75 g/kg (N1), naringin at 1.5 g/kg (N2), hesperidin at 0.75 g/kg (E1), hesperidin at 1.5 g/kg (E2) and a-tocopheryl acetate at 0.2 g/kg (E). At 42 days of age, 10 chickens per treatment group were slaughtered for meat quality and oxidative stability assessment. No significant differences were observed among groups in final body weight, carcass weight and internal organs weights (P>0.05) apart from liver that decreased linearly with increased levels of naringin (P-linear<0.05). Regarding the breast meat quality parameters, only redness (a*) value was higher in E1 and N1 group compared to VE group (P<0.05), while all the others i.e. shear values (N/mm^2^), pH_24_, cooking loss (%) and L* and b* color parameters were not significantly different among groups (P>0.05). Measurement of lipid oxidation values showed that after hesperidin and naringin dietary supplementation, malondialdehyde values decreased in tissue samples in a dose depended manner (P-linear<0.05). In conclusion, hesperidin and naringin, positively influence meat antioxidative properties without negative implications on growth performance and meat quality characteristics in poultry, thus appearing as important additives for both the consumer and the industry.

## Introduction

Poultry meat has many desirable nutritional characteristics such as low intramuscular fat content and relatively high concentrations of polyunsaturated fatty acids (PUFA) [1]. However, increasing the degree of unsaturation of muscle membranes triggers the susceptibility of poultry meat to oxidative peroxidation, leading to deterioration of meat color, odor, texture, flavor and nutritional value [2]. Oxidation by free radicals is one of the primary mechanisms of quality diminution in foods and especially in meat products [3]. Oxidation of lipids is initiated in the highly-unsaturated fatty acid fraction of membrane phospholipids, leading to the production of hydroperoxides, which are susceptible to further oxidation or decomposition to secondary reaction products such as short-chain aldehydes, ketones and other oxygenated compounds. These compounds may adversely affect lipids, pigments, proteins, carbohydrates, vitamins and the overall quality of products by causing loss of flavor, color and nutritive value and limiting shelf-life [4, 5].

The last decade, considerable interest has arisen in the use of natural antioxidants such as tocopherols, carotenoids, flavonoids, phenolic acids that could serve as alternatives to synthetic supplements for improving meat quality, without leaving residues in the product or the environment [6]. As a result, there is a strong tendency towards isolating organic antioxidants from natural sources for the protection of animal health and their products against oxidation [7]. The use of natural antioxidants can extend the shelf life and increase the acceptability of meat during retail display [8]. Nutritional approaches may be more effective than direct addition of the antioxidant to the muscle food since the compound is preferably and uniformly deposited where it is most needed [9]. Antioxidant effects of a-tocopheryl acetate (vitamin E) supplementation on chicken muscle are well established [10, 11, 12]. Dietary supplementation of the lipid soluble antioxidant vitamin E allows uniform incorporation of tocopherol into the subcellular membranes where it can effectively inhibit the oxidative reactions at their localized sites and improve meat quality due to delayed lipid oxidation and muscle discoloration compared to exogenously added antioxidants. Within tissues and after its hydrolization in the gastrointestinal tract, a-tocopheryl is localized in the highly unsaturated phospholipid bilayer of the cell membranes where it inhibits lipid oxidation by functioning as a free-radical scavenger [8, 13].

Recent years have seen a growing interest on the part of scientific community and agro-food industry into the revaluation and application of co-products in general and those obtained from citrus fruits in particular for addition to animal diets. Bioflavonoids, such as hesperidin and naringin are abundant in citrus pulp, an inexpensive by-product of citrus cultivation. Citrus pulp is derived after extraction of the juice from citrus fruits and therefore is a mixture of peel, inside portions and culled fruits of the citrus family. It has been extensively used in feeding of animals (especially ruminants) with a positive effect on preventing problems from its disposal into the environment and the necessity for costly waste management programs. Fibers from citrus fruits have an additional advantage over dietary fibers from other sources due to the presence of associated bioactive compounds (i.e. flavonoids) and could be used as functional ingredients, since they can interact physiologically to provide numerous health benefits.

Flavonoids usually contain one or more aromatic hydroxyl groups, which actively scavenge free radicals and are responsible for the antioxidant activity. They are widely distributed in the plant kingdom as secondary metabolites synthesized for defense against infection and stress conditions, such as ultraviolet light, pathogens and physical damage [14]. Flavonoids and especially their subgroup flavanones, which contain hesperidin and naringin, well known for their antioxidant properties, are health-promoting molecules with multifunctional biological activities; they have been shown to attenuate inflammation, to quench active oxygen species and their intake appears to be inversely related to risk of cardiovascular disease and several form of cancer [15]. The objective of this study was the evaluation of the effects of hesperidin or naringin dietary supplementation on broilers performance, carcass and meat quality characteristics.

## Material and Methods

### Animal management and experimental design

Two hundred and forty 1-day-old Ross 308 broiler chickens, obtained from a commercial hatchery, as hatched, were housed in a controlled environment. The birds were reared in 12 pens, of a surface area of 2 m^2^ each, until 42 d of age. Each chicken was individually wing tagged, feather-sexed and weighed on arrival. The lighting program consisted of 23L:1D on arrival, and was decreased to 18L:6D at day 7, remained constant until day 35, and thereafter gradually increased to 23L:1D at slaughter. Environmental conditions and management practices were in accordance to standard Ross guidelines. Feed, in mash form, and water were provided *ad libitum*. The composition and the chemical analysis of the three diets offered, throughout the rearing period (starter, grower and finisher), are presented in Table 1.

**Table 1:** Ingredients and chemical composition of the diets used.

Ingredients g/kg	Starter, 1 to 10 day	Grower, 11 to 22 day	Finisher, 23 to 42 day
Maize	250	200	120
Wheat	350	420	520
Soybean meal, 48% CP	325	300	275
Soybean oil	30	22	20
Vegetable fat	0	20	30
Limestone	14	13	13
Monocalcium phosphate	14	12	10
Sodium chloride	2	2	2
Sodium bicarbonate	2	2	2
Lysine	3	2.5	1.8
Methionine	3.2	2.4	2.1
Threonine	1.2	0.5	0.2
Natugrain® wheat	0.1	0.1	0.1
Phytase, Natuphos®	0.1	0.1	0.1
Antioxidant	0.12	0.12	0.12
Choline	0.7	0.7	0.7
Acidifier	2	0	0
Coccidiostat, Clinacox® 0.5%	0.2	0.2	0.5
Vitamin and mineral premix^1^	2.5	2.5	2.5
Chemical composition^2^ g/kg			
Metabolizable energy (Mj/kg)	12.80	13.22	13.64
Dry matter	884	883	883
Ash	58	51.6	50.4
Crude protein	225	210	200
Fat	52	60	67
Fiber	28.3	27.8	27.1
Lysine	14.5	12.5	11
Methionine + cystine	10.6	10.3	9.5
Calcium	10	9	8.5
Phosphorus	8	7.5	7

^1^ The vitamin and mineral premix provided per kg of diet: 13,000 IU of vitamin A (retinyl acetate), 5,000 IU of cholecalciferol, 80 mg of vitamin E (DL-α-tocopheryl acetate), 4 mg of menadione, 4.2 mg of thiamine, 8 mg of riboflavin, 6 mg of pyridoxin, 20 μg of cobalamin, 75 mg of nicotinic acid, 18 mg of pantothenic acid, 2 mg of folic acid, 240 μg of biotin, 10 mg of vitamin C (ascorbic acid), 500 mg of choline chloride, 0.23 mg of Co, 1.2 mg of I, 0.35 mg of Se, 50 mg of Fe, 140 mg of Mn, 25 mg of Cu, and 115 mg of Zn.

^2^ Calculated

The total of 240 broiler chickens where equally divided into 6 treatment groups and were allocated into 2 pens for each treatment group. Each pen contained 20 chickens. The six treatment groups were: N1 and N2, supplemented with 0.75 and 1.5 g of Naringin (Alfa Aesar GmbH & Co KG, Germany) per kg of feed respectively, E1 and E2, supplemented with 0.75 and 1.5 g of Hesperidin (TSI Europe NV, Belgium) per kg of feed respectively, control (C) with no feed additive and VE supplemented with 0.2 g a-tocopheryl acetate (vitamin E) (DSM Nutritional Products Hellas, Greece) per kg of feed, which served as positive control. Feed additives were supplemented from the 11^th^ day of age until slaughter.

### Measurements

#### Body weight, carcass and internal organs weights

At the age of 42 d, 10 chickens per treatment group (5 male and 5 female), randomly chosen, were individually weighed, electrically stunned and slaughtered. Carcass, liver, heart and fat pad weights were recorded. Chicken carcasses were then chilled at 4°C for 24h, for subsequent breast meat quality assessment and oxidative stability measurement in *pectoralis major* and *biceps femoris* muscles.

#### Meat quality

pH24 was measured by insertion of pH meter electrode (HI 99163 MeatpH Temperature Meter, Hanna instruments, Romania), calibrated at pH 4.0 and pH 7.0, into the right *pectoralis major* muscle 24 h after slaughter.

Meat color was also measured (three measurements per sample) on right *pectoralis major* after exposure to the air at room temperature for 30 min with a Miniscan XE (HunterLab, Reston, VA) chromameter set on the L* (lightness), a* (redness) and b* (yellowness) system. White and black tiles were used for calibration.

Cooking loss and shear force value were also determined in the right *pectoralis major* muscle that was dissected, weighed, placed into a thin walled plastic bag and cooked in a water-bath at 80°C for 30 min. Each sample was then cooled under tap water and equilibrated at room temperature. The muscle was weighed again for determination of cooking loss (%). Shear force was evaluated by cutting two 1.9-mm-wide x 10mm x 10 mm strips from the center of the muscles parallel to the muscle fibers. The samples were cut perpendicular to the fiber direction using a Zwick Testing Machine Model Z2.5/TN1S (Zwick GmbH and Co, Germany) equipped with a Warner-Bratzler shear [16]. Peak force values were obtained in N/mm^2^.

Oxidative stability was assessed on the basis of the malondialdehyde (MDA), a secondary lipid oxidation product formed by the hydrolysis of lipid hydroperoxides during lipid oxidation [11, 17]. In the present study, the MDA concentration was determined in the left *pectoralis major* and *biceps femoris* muscle samples, from 6 chickens per treatment. Determination took place after storage at 4°C for 3, 6 and 9 d and -20°C for 120 d after slaughter, by using a selective third-order derivative spectrophotometric method [18]. Derivative spectrophotometry was chosen over conventional because it offers improved sensitivity, specificity and reliability of the measurements, since it eliminates potential interferences from other reactive compounds. In brief, 2 g of each sample (two samples per chicken) were homogenized (Unidrive x 1000, CAT, M. Zipperer GmbH, Germany) in the presence of 8 ml aqueous trichloroacetic acid (TCA) (50 g/l) and 5 ml butylated hydroxytoluene in hexane (8 g/l), and the mixture was centrifuged for 5 min at 3000 x g. The top hexane layer was discarded and a 2.5-ml aliquot from the bottom layer was mixed with 1.5 ml of aqueous 2-thiobarbituric acid (8 g/l) to be further incubated at 70°C for 30 min. Following incubation, the mixture was cooled under tap water and submitted to third-order derivative (3D) spectrophotometry (Hitachi U3010 Spectrophotometer, Hitachi High-Technologies Corporation, Japan) in the range of 500–550 nm. The concentration of MDA (ng/g wet tissue) in the samples was determined as the height of the third-order derivative peak at 521.5 nm by referring to the standard calibration curve prepared using 1,1,3,3-tetraethoxypropane, the malondialdehyde precursor.

### Statistical analyses

Data were analyzed with SAS software [19]. ANOVA was applied for all measured traits with dietary treatment, sex and their interaction as fixed effects. Because no significant dietary treatment by sex interaction was detected sex effect was excluded from the model. Meat oxidative stability was analysed with dietary treatment, muscle type (*pectoralis major* and *biceps femoris*) and their interaction as fixed effects. The linear dose response to naringin or hesperidin was determined with contrasts among means. Pair wise comparisons with control or VE group were tested at 0.05 significance level. Results are presented as least square means ± SEM.

### Ethic statement

This study was carried out in strict accordance with the guidelines of “Council Directive 86/609/EEC regarding the protection of animals used for experimental and other scientific purposes”. The protocol was approved by the Bioethical Committee of the Agricultural University of Athens (Permit Number: 20/20032013). All efforts were made to minimize suffering.

## Results and Discussion

### Body weight, carcass and internal organs weights

Body weight (BW), carcass, heart and fat pad weights were not affected by either naringin or hesperidin dietary supplementation (P-linear>0.05, Table 2).

**Table 2:** Effect of dietary supplementation with naringin or hesperidin on broiler body, carcass and internal organ weights at the 42nd d of age (n = 10).

Antioxidant^1^	BW, g	Carcass weight, g	Dressing percentage, %	Liver, g	Liver, %	Heart, g	Heart, %	Fat pad, g	Fat pad, %
C	2814	1996	70.9	59.3	2.11	15.50	0.55	42.22	1.52
E1	2594	1847	71.2	58.6	2.26	13.29	0.51	34.62	1.33
E2	2699	1922	71.3	58.5	2.19	16.55	0.61	41.46	1.54
N1	2455	1762	71.7	49.7* ^§^	2.03^§^	13.82	0.56	29.76	1.22
N2	2673	1905	71.4	62.7	2.37	14.98	0.56	34.27	1.30
VE	2686	1906	70.9	65.8	2.47	14.34	0.54	30.88	1.12
SE	95	69	0.6	2.8	0.10	0.88	0.03	3.65	0.14
					P-linear				
C-E1-E2	0.325	0.388	0.722	0.855	0.629	0.424	0.152	0.894	0.938
C-N1-N2	0.273	0.332	0.648	0.230	0.025	0.661	0.860	0.085	0.226

^1^ C: no additive, E1 and E2: 0.75 and 1.5 g hesperidin per kg feed, respectively, N1 and N2: 0.75 and 1.5 g naringin per kg feed, respectively, VE: 0.2g a-tocopheryl acetate (vitamin E) per kg feed.

* P<0.05 compared with C group.

^§^ P<0.05 compared with VE group.

However, increased naringin levels in the diet of chickens resulted in a linear increase (P-linear<0.05) in liver percentage (% BW). The highest value for liver percentage was observed for the VE group of birds and significant difference was detected between the VE and the N1 treatment groups (P<0.05). In a previous study, no effect of hesperidin dietary supplementation (1.5–3.0 g/kg) on broilers’ final body weight (kg), body weight gain (g), feed conversion ratio, carcass and internal organs weight (g) was observed [20]. No significant effect of dietary ascorbic acid and citric flavonoids (quercetin and rutin) mixture (0.25, 0.5 or 1 g/kg) on broilers’ growth performance and carcass traits has also been reported [21]. On the other hand, a significant improvement in body weight and feed/gain ratio was recorded after hesperidin incorporation into broiler diets at the level of 20 mg/kg [22]. In the same study, a positive effect of genistein or a mixture of genistein and hesperidin (1:4) at the level of 5 mg/kg on hot carcass weight was shown and combinations of genistein and hesperidin supplementation (5 mg genistein/kg of feed or 20 mg hesperidin/kg of feed or a mixture of genistein and hesperidin (1:4) in doses of 5, 10 or 20 mg/kg of feed) improved body weight gain up to 11.3% in the finisher period, although overall feed conversion ratio (0–42d) was not influenced [22]. Similarly, dietary supplementation with *Lonicera japonica* or *Chelidonium Majus* extracts (0.2%) that contain naringin at a level of 12.16 and 7.75 mg/kg, respectively, increased broilers’ final body weight and daily weight gain [23]. Furthermore, chickens consuming a mixture of essential oil from oregano, anis and citrus peel at a level of 125 ppm appeared to have an improved feed conversion ratio compared to controls [24]. Finally, no significant differences were observed in liver, heart, gizzard and abdominal fat weight after hesperidin dietary supplementation at the level of 1.5–3.0 g/kg [20]. At the same time, no differences were observed for gizzard, liver, pancreas, large and small intestine weight after the dietary supplementation with an essential oil extract from oregano, cinnamon and pepper [25]. The controversy within the literature may arise from differences in the function of each additive and variation in weight, genetics, or management of animals in the various reported studies.

### Meat quality and oxidative stability

#### pH24 values

No effect of hesperidin or naringin dietary supplementation on pH_24_ value was observed (Table 3).

**Table 3:** Effect of dietary supplementation with naringin or hesperidin on broiler *pectoralis major* instrumental quality traits (n = 10).

Antioxidant^1^	L*	a*	b*	Cooking loss (%)	Shear force N x 100/mm^2^	pH_24_
C	55.6	5.81	14.1	14.4	8.23	5.40
E1	56.2	6.03^§^	14.4	17.1	8.51	5.38
E2	54.8	5.30	13.2	16.5	8.33	5.41
N1	54.4	5.83^§^	13.9	16.9	9.63	5.40
N2	56.8	5.09	13.7	15.6	7.77	5.41
VE	54.4	4.65*	13.7	15.5	9.92	5.41
SE	0.7	0.29	0.4	0.9	0.69	0.03
				P-linear		
C-E1-E2	0.390	0.238	0.161	0.104	0.912	0.780
C-N1-N2	0.241	0.139	0.424	0.369	0.648	0.825

^1^ C: no additive, E1 and E2: 0.75 and 1.5 g hesperidin per kg feed, respectively, N1 and N2: 0.75 and 1.5 g naringin per kg feed, respectively, VE: 0.2g a-tocopheryl acetate (vitamin E) per kg feed.

L* = lightness.

a* = redness.

b* = yellowness.

* P<0.05 compared with C group.

^§^ P<0.05 compared with VE group.

No significant difference in pH_24_ of broiler breast meat was also found after hesperidin dietary supplementation at the level of 1.5–3.0 g/kg [20]. The same results were obtained after supplementing broiler diets with oregano (3%) [12], grape seed extract (2500 ppm), green tea extract (2500 ppm) [26], cranberry extract at concentrations between 40 and 160 mg per kg feed [27], Lonicera japonica and Chelidonium Majus extracts (0.2%) [23], or a mixture of ascorbic acid and citric flavonoids (quercetin and rutin) (0.25, 0.5 or 1 g/kg) [21]. On the other hand, inclusion of a mixture of genistein and hesperidin (1:4) at the level of 20 mg/kg into broilers’ diet increased pH_45_ values (+3.1%) [22]. Contrary to results of the present study, dietary herb extract mix (mulberry leaf, Japanese honeysuckle and goldthread) supplementation increased broiler meat pH values directly after slaughter [28]. However, citrus pulp dietary supplementation (10%) appeared to decrease broiler meat ultimate pH [29].

#### Color parameters

Values of color parameters (L*, a* and b*) were not significantly influenced by hesperidin and naringin incorporation in broilers’ diets (Table 3). However, a less red colored breast meat was observed for the VE compared to C, E1 and N1 treatment groups (P<0.05). In a previous study, supplementation with hesperidin appeared not to affect color parameters of *pectoralis major* muscle in broilers [20]. Addition of a mixture of ascorbic acid and citric flavonoids (quercetin and rutin) (0.25, 0.5 or 1 g/kg) [21] or plant (grape seed or green tea) extracts [26] or a mixture of essential oil from oregano, anis and citrus peel [24] or Lonicera japonica and Chelidonium Majus extracts (0.2%) [23] did not also have any significant effect on L*, a* and b* values. Dietary herb extract mix (mulberry leaf, Japanese honeysuckle and goldthread) [28] or cranberry fruit extract [27] supplementation also did not influence breast meat color directly after slaughter. Similar results were found after citrus pulp dietary supplementation in broiler meat concerning L* and b* values [29]. However, inclusion of a mixture of genistein and hesperidin (1:4) at the level of 20 mg/kg of feed into broilers’ diet increased meat lightness (L*), without a significant effect on a* and b* values [22].

#### Shear force value–Cooking loss

Different concentrations of hesperidin or naringin supplementation did not influence the meat shear force value (Table 3). There is no evidence of naturally occurring additives dietary supplementation having a direct effect on broilers breast meat tenderness [21, 26, 27]. Cooking loss was also not significantly affected by the dietary treatment (Table 3). Incorporation of Lonicera japonica or Chelidonium Majus extracts (0.2%) did not also have any significant effect on cooking loss of breast meat in broilers [23]. No differences in meat water holding capacity were found, when an oregano supplement (3%) [12] or herb extract mix (0.3–1%) [28] or a mixture of essential oil from oregano, anis and citrus peel [24] was incorporated in broiler diet. However, combinations of genistein and hesperidin supplementation (5 mg genistein/kg of feed or 20 mg hesperidin/kg of feed or a mixture of genistein and hesperidin (1:4) in doses of 5, 10 or 20 mg/kg of feed) increased water holding capacity values [22]. At the same time, water holding capacity was significantly increased after dietary supplementation with a mixture of gallic acid and linoleic acid at a level of 1% meat [30].

#### Lipid oxidation–MDA assay

MDA measurements were not different between *pectoralis major* and *biceps femoris* muscles nor muscle type by dietary treatment interaction was significant (P>0.05) (Table 4).

**Table 4:** Effect of dietary supplementation with naringin or hesperidin on broilers’ meat (pectoralis major and biceps femoris) oxidative stability during storage (ng MDA/g meat) (n = 6).

Antioxidant^1^	Storage time^2^, days			
	3	6	9	120
C	5.70	12.16	20.54	16.62
E1	5.33	10.10^§^	17.00^§^	14.18* ^§^
E2	6.75^§^	8.06*	15.92^§^	13.71* ^§^
N1	5.79	9.03* ^§^	18.45^§^	12.13*
N2	6.84^§^	8.83*	13.15*	13.07* ^§^
VE	4.51	6.64*	8.69*	10.31*
SE	0.61	0.77	1.92	0.70
		P-value		
Antioxidant	0.078	0.002	0.001	<0.001
Muscle type	0.582	0.209	0.604	0.510
Muscle type x antioxidant	0.771	0.7219	0.994	0.417
		P-linear		
C-E1-E2	0.244	0.001	0.138	0.017
C-N1-N2	0.185	0.011	0.007	0.001

^1^ C: no additive, E1 and E2: 0.75 and 1.5 g hesperidin per kg feed, respectively, N1 and N2: 0.75 and 1.5 g naringin per kg feed, respectively, VE: 0.2g a-tocopheryl acetate (vitamin E) per kg feed.

^2^ stored for 3, 6 and 9 d at 4°C and for 120 d at -20°C.

* P<0.05 compared with C group.

^§^ P<0.05 compared with VE group.

MDA, Malondialdehyde.

Oxidative stability of meat from *pectoralis major* and *biceps femoris* muscle stored for more than 6 d appeared to improve when broilers fed diets supplemented with naringin or hesperidin. As shown in Table 4, MDA had lower values in naringin and hesperidin groups compared to controls after 6 d of storage. A linear dose response was detected (P-linear<0.05) for both flavonoids after storage for 6 and 9 d at 4°C and for 120 d at -20°C. The only exception was for the hesperidin group at 9 d of storage when no linear dose response was observed (P-linear>0.05), although lower (but not statistically significant) MDA values for E1 and E2 groups were obtained in comparison with the C group of birds. At 3 d of storage, rate of lipid oxidation was not affected by dietary supplementation with either naringin or hesperidin (P-linear>0.05). Although, the inhibition of lipid oxidation in broiler meat after dietary supplementation with hesperidin and naringin was possibly the result of their properties, the bioavailability of hesperidin and naringin cannot be directly demonstrated, because adequate analytical methodology for identification and quantification of these substances and their metabolites is not yet available for poultry tissues.

The purpose of introducing the VE treatment group in the experimental design of our study was to compare vitamin E, known for its antioxidant properties in broiler meat [10, 11, 12], with hesperidin or naringin. The lowest MDA values were detected for the vitamin E dietary supplemented broilers (Table 4). Significant differences were detected during storage for 6 or more days between the VE and C group, as expected, between the VE group and both levels of hesperidin (P<0.05), except from the E2 group at 6 days of meat storage. Concerning the naringin supplemented groups, significant differences were observed between the VE and N1 group during refrigerated storage (6 and 9 days at 4°C) and N2 group at 120 d of storage. Therefore, it can be concluded that antioxidant capacity of naringin was comparable to that of vitamin E, whereas hesperidin exerted less intense antioxidant properties compared to vitamin E.

Antioxidant activity is affected by the lipophilic properties and the chemical structure of the molecules. The phenolic OH groups act as hydrogen donors to the peroxy radicals produced during the first step in lipid oxidation, thus retarding the hydroxyl peroxide formation. It would appear that the supplements act as effective free radicals scavengers and influence the *in vivo* antioxidant defense systems such as superoxide dismutase, glutathione peroxidase and vitamin E [8]. According to previous studies, flavonoids can take over the role of a-tocopheryl acetate as a chain-breaking antioxidant in liver microsomal membranes [31]. Antioxidant dietary supplementation has been proved to be a simple and convenient strategy to uniformly introduce a natural antioxidant into phospholipid membranes where it may effectively inhibit the oxidative reactions at their localized sites [13]. It could be hypothesized that chelating properties of polyphenols would be responsible for changes in Fe and Cu content of the muscles leading to decrease MDA formation. Therefore, although flavonoids and vitamin E have a similar effect on MDA values, mechanisms of protective effects of flavonoids against MDA accumulation in muscles could be completely different from those described for vitamin E [32].

According to the findings of a previous study, hesperidin dietary supplementation improves the oxidative stability of broiler meat [20]. Beneficial results with respect to broiler meat oxidative stability were also obtained by dietary supplementation of combinations of genistein and hesperidin (5 mg genistein/kg of feed or 20 mg hesperidin/kg of feed or a mixture of genistein and hesperidin (1:4) in doses of 5, 10 or 20 mg/kg of feed) [22]. Serum superoxide dismutase level increased by the flavonoids hesperitin and naringenin dietary supplementation; at the same time, total antioxidant activity and scavenging superoxide ability were enhanced, and serum TBARS level was decreased by naringin supplementation in laying hens [33]. Serum total antioxidant capacity was improved and MDA values were depressed after combinations of genistein and hesperidin supplementation (5 mg genistein/kg of feed or 20 mg hesperidin/kg of feed or a mixture of genistein and hesperidin (1:4) in doses of 5, 10 or 20 mg/kg of feed) [34]. Similar results were reported for naringin in rabbits [35], rats [36] and mice [37]. Several reports have shown that extracts of rosemary and sage [38], tea catechins [39, 40], oregano essential oil [17] or oregano herb [41] or mixture of gallic acid and linoleic acid [30] or thymol and carvacrol [42] or quercetin [43] improved the oxidative stability of stored chicken meat when incorporated in diets. Similar reduction of meat lipid oxidation has been reported after storage at 4°C in turkeys [44] and rabbits [45] after oregano essential oil dietary supplementation (200 and 100–200 mg/kg, respectively). Increased antioxidative status in the living animal and a following increased oxidative stability of the raw product is considered beneficial for both the consumer and the processing industry.

## Conclusions

The dietary administration of hesperidin and naringin exerted significant effect on broiler breast and thigh meat antioxidative capacity, possibly indicating that these bioflavonoids were introduced into the cell phospholipids membranes in broiler muscles. The retardation of lipid oxidation rate detected in broiler meat positively affects meat shelf life with advantages for both the poultry meat industry and the consumer. However, further research is warranted to elucidate their exact action and evaluating the efficiency of citrus pulp as a dietary agent that may increase broilers' meat oxidative stability and quality since it is the main source of the naturally occurring antioxidants hesperidin and naringin.
